# Do Mediators Linking Childhood Conditions to Late-Life Chronic Inflammation Vary by Race?

**DOI:** 10.1093/geroni/igaa057.1494

**Published:** 2020-12-16

**Authors:** Kenneth Ferraro, Patricia Morton

**Affiliations:** 1 Purdue University, West Lafayette, Indiana, United States; 2 Wayne State University, Detroit, Michigan, United States

## Abstract

Recent findings suggest that childhood exposures can lead to chronic inflammation decades later, but the mechanisms underlying this relationship are relatively unknown. We investigate how childhood exposures influence adult chronic inflammation (measured by C-reactive protein) and examine five potential mediators comprising two midlife domains: socioeconomic status (SES) and health lifestyles. Using a sample of 8,891 adults aged 51 and older from the Health and Retirement Study (HRS), the analysis tests whether these life course mediators operate differently for Black, White, and Hispanic Americans. Among the six childhood domains examined, low SES and risky parental behaviors predict adult chronic inflammation, but adult health lifestyles mediate the effects of childhood SES and parental behavior. Adult SES also mediates the effect of childhood SES. Smoking and wealth exert stronger direct and indirect effects on adult inflammation for White Americans compared to Black Americans whereas BMI and exercise exert stronger direct and indirect effects for White Americans compared to Hispanic Americans. Although education mediated the effect of childhood SES on adult chronic inflammation, its effects did not vary by race. These results demonstrate that the physiological consequences of childhood exposures are carried into late-life via adult lifestyle factors and SES. In addition, the life course antecedents of chronic inflammation are distinct for Black, White, and Hispanic Americans. Future research investigating the early origins of adult health should consider not only multiple midlife mechanisms but also how resource mediation varies by race and ethnicity.

